# Peer Review for “Thyroid Hyperplasia and Neoplasm Adverse Events Associated With Glucagon-Like Peptide-1 Receptor Agonists in the Food and Drug Administration Adverse Event Reporting System: Retrospective Analysis”

**DOI:** 10.2196/59120

**Published:** 2024-05-01

**Authors:** Anonymous

**Keywords:** monotherapy, GLP, FDA, adverse, reporting, thyroid, cancer, oncology, cancers, neoplasm, neoplasms, drug, drugs, pharmacy, pharmacies, pharmacology, pharmacotherapy, pharmaceutic, pharmaceutics, pharmaceuticals, pharmaceutical, medication, medications, glucagon-like peptide-1, Food and Drug Administration

*This is the peer-review report for “Thyroid Hyperplasia and Neoplasm Adverse Events Associated With Glucagon-Like Peptide-1 Receptor Agonists in the Food and Drug Administration Adverse Event Reporting System: Retrospective Analysis.”*

## Round 1 Review

### General Comments

In this manuscript titled “Thyroid Hyperplasia and Neoplasm Adverse Events Associated With Glucagon-Like Peptide-1 Receptor Agonists in the Food and Drug Administration Adverse Event Reporting System: Retrospective Analysis” [1], the authors analyzed over 18 million reports from the Food and Drug Administration (FDA) Adverse Event Reporting System, among which over 17,000 cases were identified to have increased possibility of thyroid hyperplasia and neoplasm when taking glucagon-like peptide-1 (GLP-1) receptor agonist (RA) monotherapy. The data were compared to the cases where the patients were taking sodium-glucose cotransporter-2 (SGLT-2) inhibitor monotherapy. Please see my suggestions and concerns below.

### Suggestions and Concerns

1. The authors compared data between GLP-1 RA monotherapy and SGLT-2 inhibitor monotherapy. It would be great to have a little bit more introduction or description for the readers to understand why they compared the data with SGLT-2 inhibitor monotherapy. What is the function of SGLT-2 inhibitors during the therapy? I realized that the authors described this a bit in the *Methods* section, but more information would be appreciated to be added in the *Introduction* section for the readers to understand the rationale.
2. In Table 1, how are the unique individual thyroid hyperplasia and/or thyroid neoplasm–related adverse event (AE) reports being counted? Was it the sum of the cases number searched by the above AE Preferred Term in each section, or it was counted through searching a specific AE Preferred Term?
3. In the reporting odds ratio (ROR) analysis, where ROR = (a/b) / (c/d), I assume a indicates the number of AE cases in the exposed group, b indicates the number of non-AE cases in the exposed group, c indicates the number of AE cases in the control group, and d indicates the number of non-AE cases in control group. Is this correct? For calculating the ROR for all GLP-1 RAs (n=17,653; number of AEs=191) compared to the SGLT-2 inhibitor (n=14,102; number of AEs=7), maybe I am wrong, but should the ROR = (191 / [17,653 − 191]) / (7 / [14,102 − 7]), where (17,653 − 191) and (14,102 − 7) are the non-AE cases, which equals to 22.02? Did the authors use all the cases instead of the non-AE cases for the calculations? The same applies to the other ROR numbers and 95% CIs.
4. The authors claimed that GLP-1 RA monotherapy reports manifested a statistically significant increase in thyroid hyperplasia and neoplasm AEs when compared to SGLT-2 inhibitors. How was the statistical significance determined? Was it because the calculated ROR is over 1 (or 10) or the interval (ROR [−], ROR [+]) is large?
5. Figure 1’s resolution seems low in the document.

## Abbreviations

AE: adverse event
FDA: Food and Drug Administration
GLP-1: glucagon-like peptide-1
RA: receptor agonist
ROR: reporting odds ratio
SGLT-2: sodium-glucose cotransporter-2
